# Pelagic occurrences of the ice amphipod *Apherusa glacialis* throughout the Arctic

**DOI:** 10.1093/plankt/fbz072

**Published:** 2020-01-10

**Authors:** Erin H Kunisch, Bodil A Bluhm, Malin Daase, Rolf Gradinger, Haakon Hop, Igor A Melnikov, Øystein Varpe, Jørgen Berge

**Affiliations:** 1 Department of Arctic and Marine Biology, Faculty of Biosciences, Fisheries and Economics, UiT The Arctic University of Norway, PO Box 6050 Langnes, N-9037, Tromsø, Norway; 2 Norwegian Polar Institute, Fram Centre, PO Box 6606, N-9296, Tromsø, Norway; 3 P.P. Shirshov Institute of Oceanology, IORAS, 36 Nakhimovskiy pr., Moscow 117997, Russia; 4 The University Centre in Svalbard (UNIS), PO Box 156, 9171 Longyearbyen, Norway; 5 Norwegian Institute for Nature Research, PO Box 5685, Torgarden, NO-7485 Trondheim, Norway; 6 The Norwegian University of Science and Technology (NTNU), NO-7491 Trondheim, Norway

**Keywords:** Arctic, sea ice fauna, life history, pelagic, spatial and temporal scales, *Apherusa glacialis*

## Abstract

*Apherusa glacialis* is a common, sea ice-associated amphipod found throughout the Arctic Ocean and has long been considered permanently associated with the sea ice habitat. However, pelagic occurrences of *A. glacialis* have also been reported. It was recently suggested that *A. glacialis* overwinters at depth within the Atlantic-water inflow near Svalbard, to avoid being exported out of the Arctic Ocean through the Fram Strait. This study collated pelagic occurrence records over a 71-year period and found that *A. glacialis* was consistently found away from its presumed sea ice habitat on a pan-Arctic scale, in different depths and water masses. In the Svalbard region, *A. glacialis* was found in Atlantic Water both in winter and summer. Additionally, we analyzed *A. glacialis* size distributions throughout the year, collected mostly from sea ice, in order to elucidate potential life cycle strategies. The majority of young-of-the-year *A. glacialis* was found in the sea ice habitat during spring, supporting previous findings. Data on size distributions and sex ratios suggest a semelparous lifestyle. A synchronous seasonal vertical migration was not evident, but our data imply a more complex life history than previously assumed. We provide evidence that *A. glacialis* can no longer be regarded as an autochthonous sympagic species.

## INTRODUCTION

Polar marine ecosystems are characterized by low water temperatures and sea ice presence, which further influence biological processes. Additionally, extreme seasonality (Leu *et al*., 2015) and the unique species association with sea ice have led to distinct life history adaptations within a seasonally disparate environment. For example, Arctic copepods, such as *Calanus glacialis*, are able to time offspring release and also mature in the photic zone when food resources are optimal and subsequently overwinter at depth in diapause (Søreide *et al*., 2010; Varpe, 2012). Sea ice itself is a unique habitat for a wide range of species assemblages that live within brine channels and at the ice–water interface (Bluhm *et al*., 2010, 2018). Ice-associated (sympagic) amphipods are strongly linked to Arctic sea ice habitat, though how they overwinter and survive periods of low food resources is currently unknown (Arndt and Swadling, 2006).

*Apherusa glacialis* is one of the 5 common, endemic Arctic amphipods (along with *Gammarus wilkitzkii*, *Eusirus holmii*, *Onisimus nanseni* and *O. glacialis*) found underneath sea ice with small individuals and juveniles also occurring within brine channels. With the exception of *E. holmii*, these amphipods have typically been considered permanent residents (autochthonous) of sea ice, with their entire life cycle to occur within the sea ice habitat (Gulliksen and Lønne, 1991; Lønne and Gulliksen, 1991b; Macnaughton *et al*., 2007). *A. glacialis* is considered semelparous (Poltermann, 2000; Beuchel and Lønne, 2002), likely reaching sexual maturity at 1 year. It is assumed that *A. glacialis* mate at the onset of polar night (Melnikov, 1997) and incubates its eggs over the winter, similar to other Arctic amphipods (Węsławski and Legeżyńska, 2002). Developing juveniles are released from the female marsupial pouch the following year (late winter/early spring), when sea ice algal food conditions are optimal (Melnikov, 1997). *A. glacialis* has a short life span of approximately 2 years (Beuchel and Lønne, 2002) and is numerically the most abundant when compared to the other aforementioned ice-associated Arctic amphipods (Bradstreet and Cross, 1982; Hop *et al.*, 2000; Gradinger *et al*., 2010). This 2-year life span suggests that *A. glacialis* overwinters once in its lifetime (Poltermann, 2000). However, *A. glacialis* colonizes young, first-year ice sooner than other ice amphipods (Gulliksen and Lønne, 1989), and higher abundances of *A. glacialis* in first-year ice (Bradstreet and Cross, 1982; Arndt and Lønne, 2002) implies that a horizontal movement to colonize new ice habitats.

When drifting sea ice habitat melts or is exported out of the Arctic Ocean, it is assumed that *A. glacialis* is lost to the water column, with little to no chance of survival, especially when sea ice is exported out of the Arctic Basin (Arndt and Pavlova, 2005; Hop and Pavlova, 2008). This led to the question, how are they able to maintain a viable population within this drifting and often ephemeral habitat? In early scientific reports, *A. glacialis* was in fact classified as a strictly pelagic amphipod species in the central Arctic Basin even when sea ice was present (Barnard, 1959). Several publications recorded *A. glacialis* and also *G. wilkitzkii* in open water in the Canadian Basin (Harding, 1966), Arctic Ocean (Melnikov, 1997, 1989), Greenland Sea (Werner *et al*., 1999) and Fram Strait and Svalbard area (Arndt and Pavlova, 2005). It has earlier been suggested that *A. glacialis* employs a vertical migration strategy (Melnikov, 1989) and that *G. wilkitzkii* is able to overwinter in shallow benthic habitats (Poltermann, 1998; Arndt *et al*., 2005).

In January 2012 near the Svalbard Archipelago, *A. glacialis* was found in all net tows (*n* = 4) in deep water between 200 and 2000 m (Berge *et al*., 2012), which resulted in the introduction of a conceptual model that *A. glacialis* could potentially occupy habitats other than sea ice. *A. glacialis* was found in warmer subsurface water originating from the Atlantic Ocean (Berge *et al*., 2012). Northward flowing Atlantic Water near Svalbard contributes to basin-wide advection processes of surface and deep water within the Arctic Ocean and further influences the movement of Arctic zooplankton and sea ice biota (Bluhm *et al*., 2015; Wassmann *et al*., 2015; Hop *et al*., 2019). Therefore, the Berge *et al*. (2012) model suggests that a primary effect of being at depth in the Atlantic inflow area is that *A. glacialis* avoids being exported out of the Arctic Ocean, though some population loss still occurs via sea ice export through Fram Strait (Hop and Pavlova, 2008). If *A. glacialis* employed a vertical migration strategy, it would be able to re-colonize the sea ice habitat the following spring. Detaching from the sea ice habitat prior to or during the polar night could be a favorable life history strategy because food sources are scarce and predation rates would be lower at depth. Migration in the Arctic—a common phenomenon found in other Arctic zooplankton (Daase *et al*., 2013)—is from cold surface waters to warmer water at depth. Female *A. glacialis* found at depth were gravid (Berge *et al*., 2012), and warmer waters do support faster rates of egg development and maturation in mesozooplankton (McLaren, 1963). Furthermore, the sufficient lipid stores found in the deep-water *A. glacialis* (Berge *et al*., 2012) suggest an adequate energy supply for overwintering.

Here we address the open questions regarding the vertical distribution and life cycle of *A. glacialis* by using the most complete available data set of its occurrence in the water column on a pan-Arctic scale. The main question of our study was rather simple, yet fundamental for our general understanding of the life history of *A. glacialis*: how often is *A. glacialis* found in the water column? Our secondary objective was to determine if there were any seasonal patterns of *A. glacialis* at depth, further informing on their life cycle strategies.

## METHOD

### Pelagic occurrences of *A. glacialis*

#### Pan-Arctic historical data of A. glacialis

The spatial and temporal distribution of *A. glacialis* was investigated using data sets spanning the entire Arctic Ocean over a 71-year period, through accessing databases and individual records. *A. glacialis* data were extracted from existing pelagic zooplankton records compiled by the Arctic Ocean Diversity Census of Marine Life project, stored within the Ocean Biogeographic Information System (www.obis.org). We additionally compiled data from published and unpublished pelagic records of *A. glacialis* within the Arctic through literature searches and directly from individual researchers (see Acknowledgments). In total, we compiled *n* = 715 confirmed the presence of pelagic records (Supplementary Table S1). ‘Records’ refer to the following 3 distinct types of data: a net haul where (i) *A. glacialis* was found in the water column, but no associated depth stratum was reported, (ii) *A. glacialis* was found within a plankton tow to the surface, or (iii) *A. glacialis* was found within a depth-stratified plankton tow. In the third case, depth-stratified data were treated as individual records (*i.e.* if 1 depth-stratified tow found *A. glacialis* at 4 discrete depths, these were treated as 4 individual records). A record may contain one or more individuals. Of these 715 records, 627 had corresponding vertical tow information. While some records contained both presence and absence, this was not indicative of all records. Because of the gaps in confirmed absence data, we included presence-only records in order to investigate if *A. glacialis* is wholly dependent on the sea ice habitat within its life. The records spanned all months of the year from 1947 through 2018, though after 5 records reported in 1947, there was a 19-year gap until 1967 and a 14-year gap from 1988 to 2001. Likely, these gaps do not represent true absences of the species in planktonic environments but rather less research efforts. Within the tow records, 15 different net types with corresponding vertical tow information were used (Supplementary Table S1). Nets differed in mesh sizes and diameter openings, and presumably in towing speeds throughout the water column, adding an unquantifiable degree of bias. Data from more common sea ice sampling using e.g. suction pumps while scuba diving (Lønne, 1988) were not included due to the pelagic focus of this study.

#### New original data from the Svalbard region

A new field sampling campaign targeted the Svalbard region close to the Berge *et al*. (2012) study. In January 2017, there was a focused effort to search for deep water *A. glacialis* onboard the R/V *Helmer Hanssen* between 80°N–82°N and 12°E–22°E. At 5 stations, a depth stratifying Multinet was deployed in the deep Arctic basin down to 1800 m. Other nets (*n* = 80) deployed for different research objectives, including Multinets deployed in shallower layers (deepest depths ranged from 600 to 145 m) were also checked for the presence of *A. glacialis*. In summary, the following nets were used: a depth-stratified zooplankton Multinet sampler (Hydro-Bios, Kiel, Germany) equipped with 5 nets of 0.25 m^2^ aperture with mesh size of 180 or 64 μm, a WP2 (Hydro-Bios, Kiel, Germany) with 90 μm mesh, an MIK net (Method Isaac Kidd-a large ring net with 3.14 m^2^ opening and 1.5 mm mesh, transitioning to a 500 μm mesh for the bottom 1.5 m) and a Harstad pelagic trawl with an 8 mm mesh. Sea ice was not encountered during the expedition.

### Data integration and analysis

Different studies provided estimates of *A. glacialis* occurrences as individuals m^−3^, individuals m^−2^, number of individuals found, or presence only. Additionally, in some datasets, *A. glacialis* was originally calculated as abundances per 100 m^−3^, and these abundances were recalculated to abundances m^−3^ to compare with other datasets. We divided the pelagic presence records of *A. glacialis* into 3 groups. The first group consisted of *n* = 88 records with geographic position (latitude and longitude) and calendar date only. These records were included in the spatial mapping of the pelagic occurrence of *A. glacialis* on a pan-Arctic scale but were not included in additional analysis because of the lack of corresponding depth information. The second group (*n* = 506) contained geographic position, calendar date, and *A. glacialis* quantified from tows to the surface. In the literature, amphipods found within sea ice are commonly reported as individuals m^−2^ (Horner *et al*., 1992; Arndt and Swadling, 2006). In order to compare abundance estimates from pelagic tows to the surface to those quantified within sea ice, we re-calculated abundance of individuals m^−3^ by depth of the entire sampled water column and report these as individuals m^−2^. The third group (*n* = 121) additionally reported *A. glacialis* from depth-stratified tows. The uppermost Multinet sections (*i.e.* the ones that terminated at the surface) were not included in the third group but instead included within the second group (tows to the surface). *A. glacialis* found in depth-stratified tows were only reported in the depth strata they were present, and not in the depth strata they were absent. This implies that we do not have all the information on the entire tow (or where the tow began), only sections of a depth-stratified tow where depth-specific distribution for *A. glacialis* was reported. Therefore, we can only report these data as abundance m^−3^. Given that the data were not normally distributed, a Kruskal–Wallis test was used to test for differences in *A. glacialis* abundances. Data analysis was conducted using R (version 3.6), and maps were created with the PlotSvalbard package (version 0.8.5; Vihtakari, 2019).

### Hydrographic information for Atlantic Water in the Svalbard region

Recent hydrographic information (water temperature and salinity) were used to investigate the relationship between *A. glacialis* occurrences within specific water masses near Svalbard. For a subset of recent cruises in the Atlantic inflow gateway to the Arctic, hydrographic data were available from conductivity-temperature-depth (CTD) profiles (Supplementary Table S2). Data were used from January (11 casts from years 2012, 2014, 2015, 2016, 2017), May (6 casts from 2003, 2005, 2014), July (14 casts from 2004, 2011, 2013) and August (9 casts from 2010, 2014, 2016 and 2018). We used the CTD cast geographically closest to the location of a given depth-stratified zooplankton tow. Profiles of potential temperature (T) and salinity (S) were binned every meter. Based on these binned values, T-S plots were made to identify characteristic water masses in which *A. glacialis* had been found. Atlantic Water is defined as S > 34.92 and T > 2°C (Beszczynska-Möller *et al*., 2012; Walczowski, 2013; Menze *et al*., 2019).

### *A. glacialis* body sizes and sex ratios

We approached the potential life cycle strategies of *A. glacialis* by presenting a pan-Arctic synthesis of *A. glacialis* body sizes and information on when young-of-the-year was present. Previously published and unpublished body length data along with information on life stage and sex were collected from a 30-year period (1979 through 2017) and from all months of the year (Supplementary Table S3). Body size was determined as the length (mm) from the distal end of rostrum to the base of the telson. The majority of the size data (70%) were provided pre-sorted into 3 size classes (Melnikov, 1997; Poltermann *et al*., 2000; I. A. Melnikov, unpublished results; M. Poltermann, unpublished results): 1–2 mm (newly hatched), 3–6 mm (juveniles), and 7–16 mm (adults). Twelve percent of the tows to the surface were reported as ‘juveniles’ and ‘adults’ (Hopky *et al*., 1994a, b), and we assigned ‘juveniles’ to the 3–6 mm size class and ‘adults’ to the 7–16-mm size class. For the majority of measured individuals, sex ratio was also reported, while for a subset of the data, only sex ratio and not size was reported. Polar night months (November through February) were pooled because of otherwise low sample sizes. Remaining months are presented individually.

## RESULTS

### Pelagic occurrences of *A. glacialis*

**Fig. 1:**
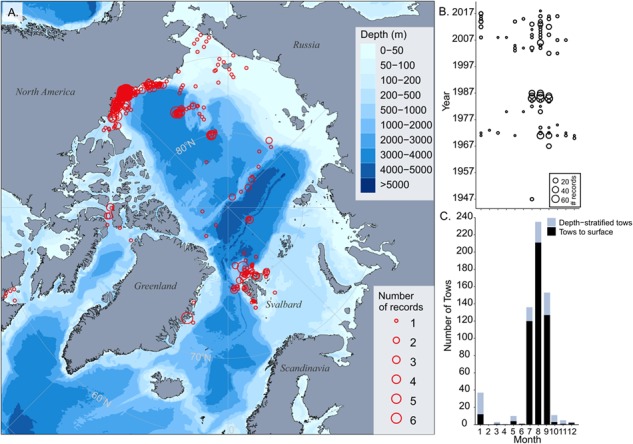
**A–C**. **A.** Spatial distribution of all pelagic records of *A. glacialis*, including records with no corresponding tow information. Circle size represents number of records within a reported geographic position. **B.** Circle size represents the same records shown in **A** but organized by month and year. **C.** All tow records (tows to the surface and depth-stratified tows) that contained the presence of *A. glacialis* within different months.

A total of 715 *A. glacialis* presence records occurred on a pan-Arctic scale between 58.7°N and 88.5°N latitude (Fig. 1A). The majority of all records (89%) were from the summer months (July, August and September, Fig. 1B), both for tows to the surface and depth-stratified tows (Fig. 1C), reflecting the generally higher research effort during the short Arctic summers. The remaining 11% of pelagic presence records were distributed over the rest of the calendar year. Some records reported counts in net hauls ranging from 1 to 1890 individuals; since most of these records also contained abundances reported per m^−2^ or m^−3^, we report them as such in order to compare with other records.

#### Tows to 0 m that contained A. glacialis

**Fig. 2:**
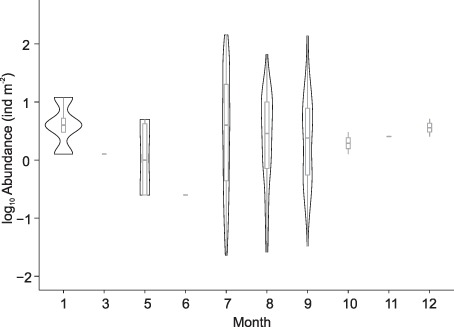
Log10 of *A. glacialis* abundance (individuals m^−2^) found in all tows to the surface. Violin plot (box shows median and interquartile range, whiskers show 95% confidence interval) within density of data (shape width depicts frequencies of values). Months 1 and 5 have only 1 whisker, due to the small ranges of *A. glacialis* abundances.

Out of 506 tows to the surface, including Multinets that terminated at the surface, 85% (430 records) were located on the Canadian Beaufort Sea shelf and slope (Fig. 1A and C). Tows started at varying depths (deepest depth ranged from 2350—2 m). When reported, total abundances of *A. glacialis* ranged from 0.023 to 143 animals m^−2^ (mean 10.2, median 2.8) Surprisingly, *A. glacialis* was consistently found within pelagic tows during the entire year (Fig. 2) but with no difference in abundance between months (Kruskal–Wallis test, *P* = 0.7). There were tows (*n* = 19) where abundance was not calculated, but the number of *A. glacialis* was reported: 1–30 individuals (mean 4.1, median 2.0). These tows were taken during January, June, July, and August.

#### Depth-stratified tows that contained A. glacialis

**Fig. 3:**
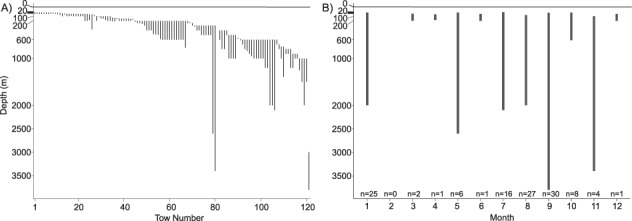
**A**. Vertical distribution of depth-stratified tows. Depth-stratified tows are individually plotted and organized by increasing shallowest depth. **B**. Minimum and maximum of depth-stratified tows, organized by month and depth (m). Total number of depth-stratified tows per month are shown.

*A. glacialis* was found in a total of *n* = 121 depth-stratified layers [*i.e.* tows that did not terminate at the surface (0 m)]. Similar to the tows to the surface, *A. glacialis* was found on a pan-Arctic scale, but in water layers mostly shallower than 1000 m (Fig. 3A). *A. glacialis* was observed in almost all months of the year, with the majority of records from January, July, August and September (Fig. 3B). Out of the 121 tows, 36% (*n* = 44) occurred within the photic zone (0—200 m) or crossed into the photic zone from a deeper depth. Abundances of *A. glacialis* in these layers (deepest depth range 382—41 m, shallowest depth range 160—13 m) were reported as: 0.004–1 m^−3^ and 1 to 3 individuals. One tow in the near surface layers quantified *A. glacialis* as 0.02 m^−2^. Seventy-five depth-stratified tows (62%) occurred within or crossed into the 200—1000 m depth layer, with abundances of 0.0004–1 m^−3^ and 1–6 individuals, respectively. *A. glacialis* occurred in 2 depth-stratified tows deeper than 1000 m with reported abundances of 0.002 m^−3^ and 2 individuals.

### *A. glacialis* in the Svalbard region

**Fig. 4:**
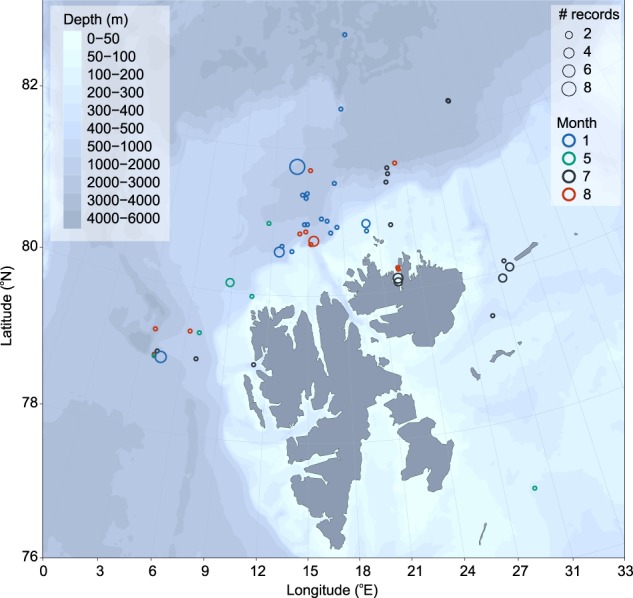
Occurrence of *Apherusa glacialis* in tows to surface and depth-stratified tows in the Svalbard region. Circle size represents the number of tows of *A. glacialis* within a reported geographic position, color represents month. Tows within this region include January (2012, 2014–2017), May (2003, 2005, 2014), July (2004, 2011, 2013) and August (2010, 2014, 2016, 2018). Bathymetry is derived from IBCAO v3.0 500-m RR grid.

**Fig. 5:**
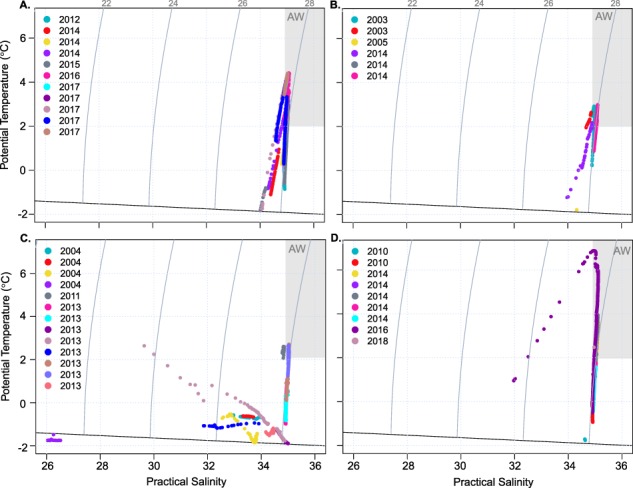
**A–D**: Occurrence of *A. glacialis* in all tows in the Svalbard region related to available CTD salinity and temperature profiles. Boxes indicate approximate position of Atlantic water. TS-diagrams of water depths where *A. glacialis* were found in **A.** January, **B.** May, **C.** July and **D.** August. For information on the full-depth profiles (*i.e.* the depths where *A. glacialis* was not found, please see Supplementary Fig. 1).

During the 2017 *Polar Night* cruise, *A. glacialis* was present in 7 out of 20 tows to the surface between 80°N and 82°N. Tows to the surface started from 400 to 100 m, and within these 7 tows, we collected a total of 54 individuals. Within 2 Multinet layers (800—400 m), we found 2 *A. glacialis*. All animals found were alive, including 8 gravid females and 1 female with an empty brood pouch. Regarding pelagic occurrences coupled with hydrographic information around the Svalbard region, *A. glacialis* was found on the shelf, inside the Arctic Rijpfjorden, as well as off-shelf both west and north of the Svalbard Archipelago (Fig. 4). *A. glacialis* was found in Atlantic Water (> 2°C) during all months of the year, regardless of location, but was also found in other water masses (Fig. 5). There was no obvious pattern of overwintering at depth in Atlantic Water.

### *A. glacialis* body sizes and sex ratios

**Fig. 6:**
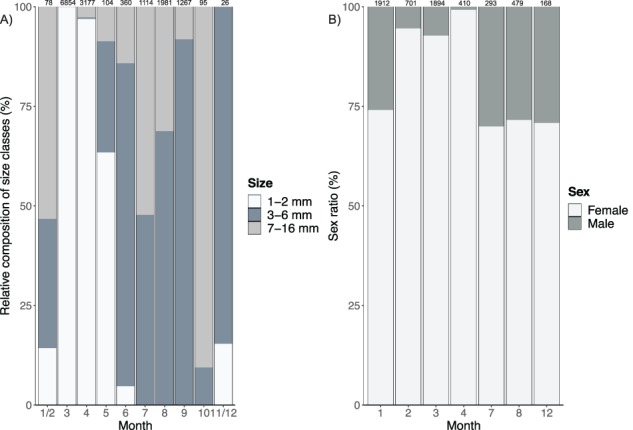
Relative contribution of (**A**) size classes and (**B**) sex ratios of *A. glacialis* across seasons, pooled across years. Size classes represent freshly hatched (1–2 mm), later juvenile (3–6 mm) and adult stages (*>* 7 mm). Records are mostly from sea ice-associated sampling. Sample numbers for each month(s) provided above bars.

We collected a total of 15 056 body size measurements, mostly from the sea ice habitat. Most researchers sampled ice-associated *A. glacialis* (88%, *n* = 13 261 individuals) directly under sea ice using scuba equipment with a plankton hand net or electric suction sampler. The remaining individuals (12%, *n* = 1 795 individuals) were collected in depth-stratified tows and tows to the surface. The smaller juvenile size class (1–2 mm) was found between November and June and was the dominating size class during the months of March through May (Fig. 6A). During the summer months in the sea ice habitat (June–September), *A. glacialis* was represented mostly by the older juvenile and adult size classes. Adults were absent in March and close to absent in April in the size data set (Fig. 6A). While there was some overlap of the measurement and sex ratio datasets, a subset of data contained only sex ratios (and no body size measurements). Compared to males, there was a much higher proportion of females found in sea ice throughout the sampled months (Kruskal–Wallis test, *P* = 0.02). Females dominated the sex ratio at all times of the year (Fig. 6B), contributing 70% during summer (July and August) and winter (December and January) and over 90% in late winter/early spring (February through April). All adults found in January [from Berge *et al*. (2012) and the 2017 *Polar Night* cruise] were females. Interestingly, we found 1 juvenile at depth during the 2017 *Polar Night* cruise, similar to the 2012 study (Berge *et al*., 2012).

## DISCUSSION

### Pelagic occurrences of *A. glacialis*

Our results demonstrate that *A. glacialis* is consistently found in the pelagic environment on a pan-Arctic scale in areas similar to the sympagic distribution of *A. glacialis* (CAFF, 2017). We recorded pelagic occurrences both on the shelves and in the basin in both the Pacific and Atlantic sectors of the Arctic Ocean. There are noticeable gaps of pelagic occurrences within the Russian shelf seas although *A. glacialis* has been found there within the sea ice habitat (CAFF, 2017). We believe that these gaps are due to the lack of sampling or lack of reporting in the available literature and do not represent a true biological pattern. It seems likely that when there is sympagic presence of *A. glacialis*, there are also pelagic occurrences, based on the similarities of our results to known sympagic distributions (CAFF, 2017).

Our vertically integrated pelagic abundances of *A. glacialis* are on a similar order of magnitude compared to the reported ranges of abundances of *A. glacialis* in Arctic sea ice (Hop *et al*., 2000). A more detailed comparison of absolute abundances between and within habitats is not possible because of the various sampling methods applied. Different plankton nets and mesh sizes used in different habitats and habitat dimensions most likely affect both the catchability of *A. glacialis* and subsequent abundance estimates of pelagic records. Despite the gear bias, however, it is apparent that distribution is patchy in both habitats, the water column and the sea ice. The distribution of sympagic amphipods is horizontally patchy (Lønne and Gulliksen, 1991a,b; Swadling *et al*., 1997), and they are often unevenly distributed among different ice features such as ridges and level ice (Arndt and Pavlova, 2005; Gradinger *et al*., 2010). In the water column, abundances were generally low, although in some tows to the surface up to hundreds of individuals were reported. Although we cannot entirely rule out that the pelagic occurrences of *A. glacialis* are due to them being lost from the sea ice habitat and that they are sinking to the sea floor, pelagic individuals occurred in areas covered by sea ice outside the melt season. Overall, our findings suggest that *A. glacialis* is not a true autochthonous species, but rather one that integrates a pelagic-sympagic coupling within its life.

We conclude that *A. glacialis* is capable of inhabiting the water column at any time of the year, even when sea ice is present. This species is rather mobile and moves between ice floes and can therefore colonize first-year ice (Lønne and Gulliksen, 1991a). No clear seasonal pattern in their pelagic occurrence was observed, although there were relatively few occurrences from the polar night compared to Arctic summer. This is in part due to the general undersampling of the polar night. Furthermore, finding *A. glacialis* both in shallower depths and in deep water during the polar night supports recent findings that many pelagic organisms maintain activity during the winter (Berge *et al*., 2015). Even though *A. glacialis* was found in deep water during periods of assumed low surface water food availability (*i.e.* polar night), they were also found in deep water during months when sympagic food sources were presumably available. For example, *A. glacialis* was found under ice and considered highly reliant on the surrounding sympagic food sources (Kohlbach *et al*., 2016) during the same summer months we find conspecifics in deep water. The overall seasonal patterns of *A. glacialis* in deep water do not follow patterns of *Calanus hyperboreus* that employs a deep overwintering migration strategy and a synchronous ascent to the surface after winter (Hirche, 1997). Our depth-stratified records with no clear seasonal pattern of *A. glacialis* occurrences suggest some plasticity in their life history strategy.

Greater plasticity in life history traits than previously assumed has also recently been documented in abundant Arctic pelagic species. Specifically regarding the paradigm that entire populations overwinter at depth, it has been found that *Calanus finmarchicus* and *C. glacialis* are also distributed throughout the water column during the polar night (Daase *et al*., 2014, 2018; Basedow *et al*., 2018). Regarding ice amphipod species, pelagic occurrences of *G. wilkitzkii, Onisimus glacialis* and *O. nanseni* in the Fram Strait at different seasons (Werner, 2006) have so far been interpreted as a potential dead end of their life cycles (Werner *et al*., 1999). However, *G. wilkitzkii* has been found alive at the sea floor in both Svalbard fjords (Arndt *et al*., 2005) and in NE Greenland (R. Fredriksen and B.A. Bluhm, pers. comm.) where ice exits the Arctic during late summer. Furthermore, *O. glacialis* females are often absent from sea ice, suggesting reproduction elsewhere (Arndt and Beuchel, 2006), and this species has been found in vertical plankton tows in deep water (Melnikov, 1997). Both *O. glacialis* and *O. nanseni* have in fact been previously described as temporary occupants of sea ice (Melnikov and Kulikov, 1980). Because *A. glacialis* is found both under sea ice and in deep water, it is possible that there could be cryptic genetic variation within discrete populations, although there is no current evidence to support this. Incorporating molecular analysis into future population studies would provide insight into this question. Regardless, it is clear that higher degrees of plasticity exist than what has been previously assumed in various Arctic crustaceans.

An added benefit of *A. glacialis* vertically migrating within the Atlantic Water inflow is that individuals would be transported back into the Arctic Ocean leading to reduced advective losses at the population scale (Berge *et al*., 2012). Surface and deep-water currents carry large volumes of warm and saline Atlantic Water into the Arctic Ocean via the Fram Strait and West Spitsbergen Current, with small amounts of Atlantic Water returning southward via bifurcation and eddy recirculation (Hattermann *et al*., 2016). The core of Atlantic Water around the northwest Svalbard archipelago is found between 75 and 500 m in epipelagic and mesopelagic water depths, thereby isolated from sea ice and the colder and fresher surface water layer (Aagaard *et al*., 1981; Beszczynska-Möller *et al*., 2011; Pérez-Hernández *et al*., 2017). Atlantic Water is also found close to or at the sea surface north of the Barents Sea (Rudels *et al*.*,* 2013; Lind *et al*., 2018). Furthermore, the speed of Atlantic Water inflow can vary both within and between seasons, which can affect the overall distribution of planktonic organisms (Hop *et al*., 2019). Berge *et al*. (2012) estimated return speeds at 2–3 months if *A. glacialis* was within the core of Atlantic water.

Within our dataset, *A. glacialis* was consistently present in both Arctic and Atlantic Waters, although there was no seasonality of where they were found when. This finding does not wholly support the hypothesis put forth by Berge *et al*. (2012), but we cannot entirely refute it either. There seems to be no overall synchronous movement within the population, though the *A. glacialis* individuals found within the Atlantic Water would have the added benefit of being transported back into the Arctic Ocean. While it is uncertain how far *A. glacialis* could be transported back into the central Arctic Ocean within their life cycle, this open question could be resolved within a particle tracking model (Doös *et al*., 2017), releasing particles at specific depths within the Arctic Ocean in scenarios with and without sea ice.

**Fig. 7:**
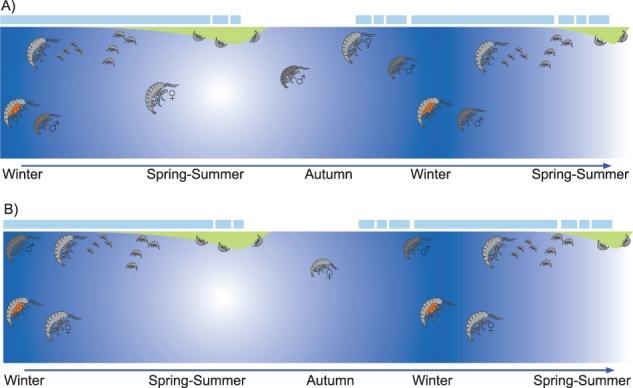
Conceptual illustrations of proposed life history strategies of *A. glacialis*, for one calendar year. Light blue indicates ice cover, green indicates ice algal bloom and blue shading indicates seasonality. **A**. A scenario where adults of *A. glacialis* are distributed both under ice and in the water column year-round, and females (both gravid and those not carrying eggs) employ vertical movement. **B**. A scenario where only *A. glacialis* females (both gravid and those that are not carrying eggs) undergo a vertical migration away from sea ice during polar night.

### *A. glacialis* life history, revisited

Within our pelagic dataset, we cannot conclude whether all *A. glacialis* found at depth were in good body condition or ‘dead sinkers’. However, the few records when body condition was reported (Berge *et al*., 2012), or observed by the authors, demonstrate that all *A. glacialis* found (regardless of depth or season) were in good body condition and that some were gravid females. An intriguing result is that when we searched for *A. glacialis* in deep water during the polar night in 2017, we again found gravid females similar to the previous findings (Berge *et al*., 2012).

Information on body size and sex ratios throughout the year can provide insights into life history strategies (Varpe and Ejsmond, 2018). Melnikov (1997, 1989) concluded that reproduction of *A. glacialis* occurs during polar night, although it is unknown at what depths mating occurs. The present study supports the previous findings that juvenile offspring are likely released in late winter/early spring, evidenced by a strong increase in the number of the smallest size class found under sea ice at this time (Melnikov, 1989; Poltermann *et al*., 2000). As the summer progresses, we document a development into the next size class with the largest proportion of adults occurring during autumn. The apparent low proportion of adults found under sea ice during the winter/early spring also supports earlier findings (Werner and Auel, 2005) and coincides with adult occurrences in the water column during these same months in the present study. While it has been suggested that *A. glacialis* breeds only once during its lifetime (Melnikov and Kulikov, 1980; Poltermann *et al*., 2000), juveniles are released in successive batches through time based on our dataset and previous studies (Poltermann *et al*., 2000; Beuchel and Lønne, 2002). Thus, our findings further support that *A. glacialis* is semelparous (Varpe and Esjmond, 2018).

In all months sampled, there was a much higher proportion of females than males in the under ice habitat, although relative adult percentages were low in the spring months. Our data suggest that adult males comprise 25% of the sex ratio consistently throughout all the months but February to April, similar to previous studies of *A. glacialis* (Melnikov and Kulikov, 1980; Poltermann *et al*., 2000). One reason for this could be that amphipod males are generally underestimated because it is much more difficult to positively identify male sex organs (minute genital papillae) compared to female oostegites (Chapman, 2007). This same bias, however, would apply to other sympagic amphipods, but the *A. glacialis* sex ratio is in stark contrast to the other ice amphipods. *G. wilkitzkii* has a 1:1 sex ratio (Poltermann *et al*., 2000), while *O. nanseni* and *O. glacialis* fluctuated between 1:1 and a dominance of either males or females depending on time of year (Arndt and Beuchel, 2006). Higher male amphipod mortality (Thurston, 1972; Powell and Moore, 2007) or males having a smaller seasonal presence (for example, males having a large role during mating, but few roles outside of mating) could be the cause of skewed sex ratios observed. For *A. glacialis*, it could be that males die soon after breeding, resulting in their particular scarcity during February to April.

Linking the data on body sizes, sex ratios and pelagic occurrences, we suggest an updated conceptual model of *A. glacialis* life history (Fig. 7). We envision possibly 3 different scenarios. Based on the findings that there can be *A. glacialis* individuals anywhere at any time, it could be that adult females and males are distributed both under ice and in the water column year-round (Fig. 7A). The second scenario suggests that only females undergo a vertical migration away from sea ice, supported by our findings of gravid females in deep water during the polar night (Fig. 7B). The third scenario (not pictured) is that this species has developed a high degree of plasticity to inhabit both the sea ice and water column, though how it will adapt to ice-free summers is unknown.

## CONCLUSION

We found clear evidence that *A. glacialis* regularly occurs within the pelagic realm, during all seasons. The scant records on body condition suggest that *A. glacialis* can successfully inhabit pelagic habitats. The data provide some support to the conceptual adaptive-advection model suggested by Berge *et al*. (2012), though our data are inconclusive on whether *A. glacialis* conducts a vertical overwintering migration and if ice drift versus water current speed makes return possible within their short life cycle. Given that *A. glacialis* is relatively mobile, can successfully inhabit different under ice structures and is found in the pelagic environment on a pan-Arctic scale, we suggest that *A. glacialis* does move in and out of the sea ice habitat and can no longer be regarded solely as an autochthonous sympagic species.

While we have demonstrated that *A. glacialis* is not as dependent on sea ice as previously assumed, sea ice habitat is still a critical part of their life history strategy, evidenced by the hatching and maturation of young *A. glacialis* in the under ice habitat. Due to climate change, the decline of Arctic sea ice extent basin-wide (Stroeve and Notz, 2018) with concomitant loss of multiyear sea ice (Kwok and Rothrock, 2009; Maslanik *et al*., 2011) has the potential to trigger ecosystem-level perturbations and affects species that inhabit sea ice, including *A. glacialis*. Both multiyear sea ice and pressure ridges provide a longer lasting habitat for ice-associated organisms (Gradinger *et al*., 2010) than thinner and smoother first-year ice. As thinner and weaker ice drifts and melts faster (Zhang *et al*., 2012; Kwok *et al*., 2013) and is exposed to more wave action, this could result in the flushing of species more easily into the surrounding water. Additionally, these changes in Arctic sea ice will result in a different under ice light environment, potentially resulting in higher predation rates from visual predators (Varpe *et al*., 2015). A complete pelagic lifestyle may be more energetically demanding (Seibel and Drazen, 2007). Increases in locomotion in order to search for food, mates and avoid predators can possibly affect metabolic rates and overall fitness of *A. glacialis*.

In conclusion, the combination of the occurrence of early life stages and females within the sea ice habitat, along with ice-algal food sources making up large proportions of adult diet, suggests that *A. glacialis* will capitalize on this habitat when available. This study gives evidence, however, that *A. glacialis* does not exclusively use the sea ice habitat, allowing them to potentially adapt to future ice-free scenarios. Therefore, a more comprehensive understanding is needed of its life history and how presence away from sea ice contributes to their overall strategy. Plasticity among organisms inhabiting under ice habitat may be an adaptive trait allowing populations to sustain themselves in an ephemeral sea ice habitat. Knowing that other Arctic ice amphipods can also occur away from sea ice, future research could investigate this phenomenon in more regional or seasonal detail, especially in areas with pronounced sea ice loss.

## Supplementary Material

supplementary_table_1_records_v2_fbz072

supplementary_table_2_ctd_sources_fbz072

supplementary_table_3__records_v2_fbz072

Supp_Fig1_final_fbz072
