# Supplementary material for: Pelagic occurrences of the ice amphipod Apherusa glacialis throughout the Arctic
Source: J Plankton Res. 2020 Jan 10;42(1):73–86. doi: 10.1093/plankt/fbz072 (PMC6994818; doi:10.1093/plankt/fbz072)
Supplement: supplementary_table_1_records_v2_fbz072 [file supplementary_table_1_records_v2_fbz072.doc]

I.

| Year | Month | N records | Region |
| --- | --- | --- | --- |
| 1947 | 7 | 5 | Ugava Bay (Hudson Strait) |
| 1967 | 9 | 16 | Arctic Ocean |
| 1970-1973 | 1-3, 5, 9-12 | 14 | Arctic Ocean |
| 1971 | 8, 9 | 41 | Beaufort Sea |
| 1971 | 8, 9 | 30 | Beaufort Sea |
| 1972 | 8, 9 | 8 | Beaufort Sea |
| 1974 | 8, 9 | 4 | Beaufort Sea |
| 1975 | 10 | 1 | Arctic Ocean |
| 1980 | 8 | 10 | Beaufort Sea |
| 1980 | 4, 6 | 2 | Beaufort Sea |
| 1982, 1984 | 7, 8 | 5 | Svalbard |
| 1984 | 7, 9 | 13 | Beaufort Sea |
| 1985 | 7, 8, 9 | 178 | Beaufort Sea |
| 1986 | 7, 8, 9 | 107 | Beaufort Sea |
| 1987 | 7, 8 | 63 | Beaufort Sea |
| 2002 | 9 | 7 | Beaufort Sea |
| 2002 | 9 | 2 | Fram Strait |
| 2002 | 9, 10 | 4 | Beaufort Sea |
| 2003 | 5 | 2 | Arctic Ocean |
| 2003, 2004 | 7, 8 | 3 | Barents Sea |
| 2004 | 7 | 9 | Arctic Ocean, Barents Sea |
| 2004 | 5, 6 | 3 | Svalbard |
| 2005 | 5 | 1 | Barents Sea |
| 2005, 2006, 2012 | 8 | 40 | Beaufort Sea |
| 2007, 2008 | 1, 3, 4, 7, 10, 11 | 12 | Beaufort Sea |
| 2009 | 9 | 5 | East Siberian Sea |
| 2009 | 7, 8 | 8 | West Ice, Greenland |
| 2009, 2012 | 8, 9 | 13 | Chukchi Sea |
| 2010, 2013 | 7, 8 | 5 | Arctic Ocean |
| 2011 | 7 | 1 | Svalbard |
| 2011 | 8 | 1 | Svalbard |
| 2012 | 1 | 8 | Arctic Ocean |
| 2013 | 7 | 8 | Fram Strait, Svalbard |
| 2013 | 9 | 1 | Beaufort Sea |
| 2011-2014 | 8, 9 | 31 | Beaufort Sea |
| 2014 | 1,5,8 | 8 | Arctic Ocean |
| 2014 | 1,5,8 | 12 | Fram Strait, Svalbard |
| 2015 | 6 | 2 | Arctic Ocean |
| 2015 | 9 | 4 | Beaufort Sea |
| 2015, 2016 | 9, 10 | 8 | Arctic Ocean |
| 2015-2017 | 1 | 14 | Arctic Ocean |
| 2016, 2018 | 8 | 6 | Arctic Ocean |

II.

| Net Type and Mesh Size | |  |
| --- | --- | --- |
| n/a | |  |
| 2 m^2^ net 215 µm | |  |
| Nansen closing net 333 µm | |  |
| Plankton net 0.5 m diameter, 573 µm | |  |
| Plankton net 0.5 m diameter, 573 µm | |  |
| Norpac net 0.25 m diameter, 570 µm | |  |
| Plankton net 73 µm, Plankton net 569 µm | |  |
| Juday net 180 µm | |  |
| n/a | |  |
| Plankton net 0.75 m diameter, 308 µm | |  |
| WP2 net | |  |
| Neuston net 500 µm, Wisconsin net 763 µm | |  |
| Bongo net 85 µm, Bongo net 500 µm, Neuston net 500 µm | |  |
| Bongo net 85 µm, Bongo net 500 µm, Neuston net 500 µm | |  |
| Bongo net 85 µm, Bongo net 500 µm, Neuston net 500 µm | |  |
| Bongo net 333 µm | |  |
| Tucker trawl 1 m^2^ diameter, 1 mm, Pelagic trawl | |  |
| Plankton net 1 m^2^, 200 µm | |  |
| Multinet 180 µm | |  |
| WP2 net 500 µm | |  |
| Multinet 180 µm | |  |
| WP2 net 180 µm | |  |
| Multinet 180 µm | |  |
| Bongo net 500 µm, Multinet 150 µm | |  |
| Multinet 200 µm | |  |
| Bongo net 505 µm | |  |
| WP2 modified net 45 µm | |  |
| Bongo net 505 µm | |  |
| Multinet, Multinet 180 µm | |  |
| Multinet 180 µm | |  |
| VanVeen grab | |  |
| Multinet 180 µm, WP3 net | |  |
| Multinet | |  |
| Multinet 150 µm | |  |
| Vertical 150 µm, Bongo 505 µm, Multinet 150 µm | |  |
| Multinet 180 µm | |  |
| Multinet 180 µm | |  |
| Rectangular midwater trawl | |  |
| Juday net 180 µm | |  |
| Multinet 150 µm | |  |
| Multinet 180 µm, WP2 net 90 μm, MIK net 1.5 mm, Pelagic trawl 8 mm | |  |
| Multinet 200 µm, Tucker trawl  III. | |  |
| Unit | Reference | |
| n/a | IOBIS, Grainger, 2011 | |
| ind./m^3^ | IOBIS, Arctic Ocean 1967 | |
| ind./m^3^ | IOBIS, Drifting Station Severnyi Polyus, Markhaseva et al., 1985 | |
| ind./m^3^ | IOBIS, McConnell, M., 1977 | |
| ind./m^3^ | IOBIS, Beaufort Sea 1971 | |
| ind./m^3^, mg/m^3^ | Western Beaufort Ecological cruise (WEBSEC), 1972 | |
| ind./m^3^ | Grainger, E. H. and C. Grohe, 1975 | |
| ind./m^3^ | K.N. Kosobokova (unpublished) | |
| ind./m^3^ | IOBIS, Lewis, 1987, Sameoto, 2013 | |
| ind./m^3^ | IOBIS, Horner, 1985 | |
| ind./sample | J.M. Węsławski (unpublished) | |
| ind./sample, mg/m^3^ | IOBIS, Hopky, G.E., M.J. Lawrence, D.B. Chiperzak, 1994a | |
| ind./sample, mg/m^3^ | IOBIS, Hopky, G.E., M.J. Lawrence, D.B. Chiperzak, 1994a | |
| mg/m^3^ | IOBIS, Hopky, G.E., M.J. Lawrence, D.B. Chiperzak, 1994b | |
| ind./sample, mg/m^3^ | IOBIS, Hopky, G.E., M.J. Lawrence, D.B. Chiperzak, 1994c | |
| ind./m^3^ | Walkusz et al., 2008 | |
| mg/m^3^ | IOBIS, Arndt 2005b | |
| ind./m^3^, ind./sample, mg/m^3^ | Darnis et al., 2008 | |
| ind./m^3^, ind./sample | Norwegian Polar Institute, A. Wold (unpublished) | |
| ind./m^3^, ind./sample | W. Walkusz (unpublished) | |
| ind./m^3^, ind./sample | Norwegian Polar Institute, A. Wold (unpublished) | |
| ind./m^3^, ind./sample | W. Walkusz (unpublished) | |
| ind./m^3^ | Norwegian Polar Institute, A. Wold (unpublished) | |
| ind./m^3^, ind./sample | W. Walkusz (unpublished) | |
| ind./m^3^, ind./sample | G. Darnis (unpublished) | |
| ind./m^3^ | E. Ershova et al., 2015 | |
| ind./sample | M. Wingding, Greenland Institute of Natural Resources (unpublished) | |
| ind./m^3^ | E. Ershova et al., 2015 | |
| ind./m^3^ | Norwegian Polar Institute, A. Wold (unpublished) | |
| ind./m^3^ | Norwegian Polar Institute, A. Wold (unpublished) | |
| ind./sample | J.M. Węsławski (unpublished) | |
| ind./sample | Berge et al., 2012 | |
| ind./m^3^ | Norwegian Polar Institute, A. Wold (unpublished) | |
| ind./m^2^ | Smoot and Hopcroft, 2017 | |
| ind./m^3^ | Beaufish and Transboundary, C. Smoot, R. Hopcroft (unpublished) | |
| ind./m^3^, ind./sample | E. Halvorsen, R/V Helmer Hanssen Carbon Bridge cruise (unpublished) | |
| ind./m^3^, ind./sample | E. Halvorsen, R/V Helmer Hanssen Carbon Bridge cruise (unpublished) | |
| ind./sample | F. Schaafsma, 2018 | |
| ind./m^3^ | NABOS, E. Ershova, K.N. Kosobokova (unpublished) | |
| ind./sample | R/V Polarstern, PS94/125, PS101/201, Kosobokova, K.N. (unpublished) | |
| ind./m^3^, ind./sample | R/V Helmer Hanssen Marine Night 2015 and 2016, Polar Night cruise 2017 (this study) | |
| ind./m^3^, ind./sample | R/V Helmer Hanssen Aug-Sept 2016, 2018 (this study) | |
