# Supplementary material for: Pelagic occurrences of the ice amphipod Apherusa glacialis throughout the Arctic
Source: J Plankton Res. 2020 Jan 10;42(1):73–86. doi: 10.1093/plankt/fbz072 (PMC6994818; doi:10.1093/plankt/fbz072)
Supplement: supplementary_table_2_ctd_sources_fbz072 [file supplementary_table_2_ctd_sources_fbz072.doc]

I.

| Date | Program name/project | Source |
| --- | --- | --- |
| May 2003 | ON THIN ICE | Norwegian Polar Institute |
| July 2004 | CABANERA | Norwegian Polar Institute |
| May 2005 | CABANERA | Norwegian Polar Institute |
| August 2010 | ICE10 | Norwegian Polar Institute |
| July 2011 | MOSJ11 | Norwegian Polar Institute |
| January 2012 | Polar Night 2012 | UiT |
| July 2013 | MOSJ_ICE13 | Norwegian Polar Institute |
| January 2014 | CARBON BRIDGE | UiT |
| May 2014 | CARBON BRIDGE | UiT |
| August 2014 | CARBON BRIDGE | UiT |
| January 2015 | Marine Night 2015 | UiT |
| January 2016 | Marine Night 2016 | UiT |
| August 2016 | UNIS AB320 | UiT |
| January 2017 | this paper | UiT |
| August 2018 | UNIS AB320 | UiT |
