# Supplementary material for: Pelagic occurrences of the ice amphipod Apherusa glacialis throughout the Arctic
Source: J Plankton Res. 2020 Jan 10;42(1):73–86. doi: 10.1093/plankt/fbz072 (PMC6994818; doi:10.1093/plankt/fbz072)
Supplement: supplementary_table_3__records_v2_fbz072 [file supplementary_table_3__records_v2_fbz072.doc]

I.

| YEAR | MONTH |
| --- | --- |
| 1979-1980 | 1-4, 12 |
| 1980 | 3 |
| 1981 | 3, 4 |
| 1985 | 7, 8, 9 |
| 1987 | 7, 8 |
| 1993 | 8 |
| 1994 | 8 |
| 1995 | 6 |
| 1996 | 7, 8 |
| 1997 | 10 |
| 1999 | 9, 10 |
| 1999 | 10 |
| 1999-2000 | 9 |
| 2000 | 8, 9 |
| 2004 | 9 |
| 2007 | 4 |
| 2007 | 4 |
| 2008 | 4 |
| 2009 | 4 |
| 2010 | 4 |
| 2011 | 4 |
| 2012 | 1 |
| 2015 | 5, 6 |
| 2016 | 8 |
| 2017 | 1 |
| 2017 | 7 |

II.

| EXPEDITION/LOCATION | DEPTH (m) |
| --- | --- |
| North Pole Drifting Ice Station-22, Canadian Arctic Basin | under ice (0 m and 5 m) |
| North Pole Drifting Ice Station-24, Arctic Basin | under ice |
| North Pole Drifting Ice Station-22, Canadian Arctic Basin | under ice |
| Beaufort Sea Shelf | tows to surface |
| Beaufort Sea Shelf | tows to surface |
| R/V Polarstern, ARK IX/4, Barents and Laptev Seas | under ice |
| Franz Josef Land | under ice |
| ICE-BAR, Barents Sea | under ice |
| ICE-BAR, Barents Sea | under ice |
| SHEBA (Surface Heat Budget of the Arctic Ocean) Drifting Ice Station, Canadian Arctic Basin | under ice |
| R/V Helmer Hanssen, Barents Sea | under ice |
| R/V Helmer Hanssen, E Greenland | under ice |
| R/V Helmer Hanssen, NE Svalbard | under ice |
| R/V Ak. Fedorov, Canadian Arctic Basin | under ice |
| R/V Helmer Hanssen, Barents Sea | under ice |
| NP 2015 | under ice |
| BARNEO Drifting Ice Camp | under ice |
| BARNEO Drifting Ice Camp | under ice |
| BARNEO Drifting Ice Camp | under ice |
| BARNEO Drifting Ice Camp | under ice |
| BARNEO Drifting Ice Camp | under ice |
| R/V Helmer Hanssen, Barents Sea | 2000-600 |
| NP 2015 | under ice |
| R/V Helmer Hanssen, Barents Sea | 1200-0 |
| R/V Helmer Hanssen, Barents Sea | tows to surface, 800-400 |
| R/V Polarstern PS106, Barents Sea and Arctic Ocean | under ice |

III.

| NUMBER A. glacialis MEASURED | NUMBER A. Glacialis SEX DETERMINATION |
| --- | --- |
| 8527 | 5069 |
| 6 |  |
| 6 | 4 |
| 135 |  |
| 1603 |  |
| 101 | 95 |
| 120 | 92 |
| 24 |  |
| 1025 | 585 |
| 41 |  |
| 103 |  |
| 60 |  |
| 14 |  |
| 1200 |  |
| 37 |  |
| 931 |  |
| 150 |  |
| 3 |  |
| 39 |  |
| 580 |  |
| 113 |  |
| 12 | 1 |
| 130 |  |
| 8 |  |
| 37 | 11 |
| 51 |  |

IV.

| SOURCE |
| --- |
| Melnikov, I. 1997 |
| Melnikov, I. 1997 |
| Melnikov, I. 1997 |
| Hopky et al. 1994a |
| Hopky et al. 1994b |
| Poltermann, M. (unpublished) |
| Poltermann et al. 2000 |
| Poltermann et al. 2000 |
| Poltermann et al. 2000 |
| Melnikov, I. (unpublished) |
| Poltermann, M. (unpublished) |
| Søreide, J. (unpublished) |
| Søreide, J. (unpublished) |
| Melnikov, I. (unpublished) |
| Berge, J. (unbpublished) |
| Melnikov, I. (unpublished) |
| Melnikov, I. (unpublished) |
| Melnikov, I. (unpublished) |
| Melnikov, I. (unpublished) |
| Melnikov, I. (unpublished) |
| Melnikov, I. (unpublished) |
| Berge et al. 2012 |
| Melnikov, I. (unpublished) |
| this study |
| this study |
| this study |
