# Supplementary figures and images for: Pelagic occurrences of the ice amphipod Apherusa glacialis throughout the Arctic
Source: J Plankton Res. 2020 Jan 10;42(1):73–86. doi: 10.1093/plankt/fbz072 (PMC6994818; doi:10.1093/plankt/fbz072)

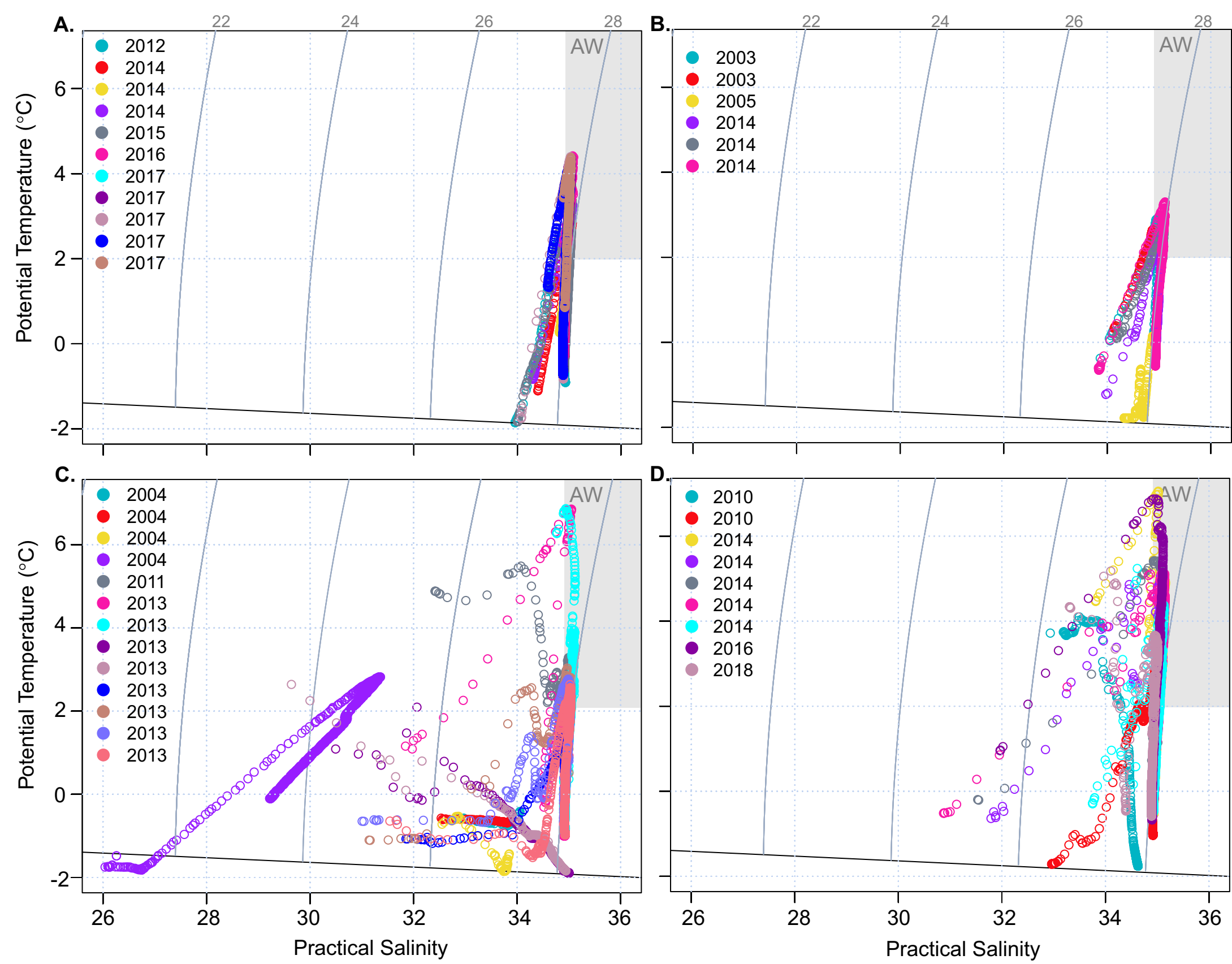

Supplement: Supp_Fig1_final_fbz072 [file supp_fig1_final_fbz072.pdf]
